# A Comparative Study of Skeletal Muscle Relaxant Effects of Thiocolchicoside, Diazepam and Their Combination in Wistar Rats Using the Rotarod Apparatus

**DOI:** 10.7759/cureus.69513

**Published:** 2024-09-16

**Authors:** Akash Devi, Chitra Khanwelkar

**Affiliations:** 1 Pharmacology and Therapeutics, Krishna Vishwa Vidyapeeth (Deemed to Be University), Karad, IND; 2 Pharmacology, Krishna Vishwa Vidyapeeth (Deemed to Be University), Karad, IND

**Keywords:** diazepam, rota rod apparatus, skeletal muscle relaxant activity, thiocolchicoside, wistar rats

## Abstract

Introduction

Thiocolchicoside (THC) is a semi-synthetic derivative of colchicoside and a naturally occurring compound in the seeds of the *Gloriosa Superba L. *plant. At present, it is used as a skeletal muscle relaxant. It is used to treat rheumatic arthritis, orthopaedic conditions, and trauma. Topically, it is used to relieve excruciating spasms in the muscles. It is also used to treat a wide range of conditions, which include surgical pain, cervicobrachial neuralgia, acute to chronic torticollis, Parkinson's disease, drug-induced Parkinsonism, spastic hemiplegia, tooth soreness, acute lower back pain, etc. THC is used in conjunction with non-steroidal anti-inflammatory drugs (NSAIDs), muscle relaxants, and analgesics. By downregulating and inhibiting the synthesis of NF-κB-regulated gene products, it possesses anti-inflammatory properties. To prevent aneuploidy, the European Medical Agency has placed restrictions on the use of THC, stating that it should not be injected for five days or taken orally for longer than seven days. This is because its metabolism in the body may lead to the production of the metabolite M2. When used during pregnancy, the growing fetus is more negatively impacted. It has also been connected to infertility in men.

Aim

To study the skeletal muscle relaxant effects of THC in Wistar rats in comparison with standard drugs.

Materials and methods

The skeletal muscle relaxant activity level was measured using the rotarod apparatus. There were five groups of Wistar rats (n = 6). Group I received 0.9% normal saline (NS) as a control. Group II (test group) received THC 2 mg/kg intraperitoneally (ip). Group III (test group) received THC 4 mg/kg (ip). Group IV diazepam (DIZ) 3 mg/kg (ip) which is the standard treatment group. Group V received a combination of THC 2 mg/kg and DIZ 3 mg/kg. After treatment, retention time was noted at intervals of 30, 60, and 120 minutes.

Results

Group I (control), Group II (THC 2 mg/kg) and Group III (THC 4 mg/kg) did not demonstrate any skeletal muscle relaxant activity. Group IV (DIZ 3 mg/kg) and Group V (THC 2 mg/kg + DIZ 3 mg/kg) showed statistically significant skeletal muscle relaxant activity when compared to control. However, there was no statistically significant difference between the skeletal muscle relaxant activity of these two groups. This indicates that the addition of THC did not potentiate the skeletal muscle relaxant activity of DIZ.

Conclusion

THC didn’t show skeletal muscle relaxant properties in both doses and did not potentiate the activity of DIZ.

## Introduction

Skeletal muscle relaxants work by inhibiting muscle tone or inducing paralysis, either at the neuromuscular junction (NMJ) located peripherally or directly at the muscle fibres, as well as centrally within the cerebrospinal axis [1]. At the NMJ, the motor neuron ceases to deliver its chemical messenger, acetylcholine (Ach), into the synapses. This causes the skeletal muscle and fibres to relax the NMJ. The sarcoplasmic reticulum (SR) gates get closed upon repolarization of the muscle fibre, thereby halting the release of calcium (Ca2+). Additionally, the sarcoplasm will be refilled with Ca2+ through adenosine triphosphate (ATP)-powered pumps. Thin filament actin-binding sites are thereby "reshielded.” The muscle fibre relaxes and releases tension when a cross-bridge between the thin and thick filaments cannot be formed [2].

Currently, thiocolchicoside (THC) is referred to as an agent that relaxes skeletal muscle. It is used in conjunction with nonsteroidal anti-inflammatory drugs (NSAIDs) for the treatment of rheumatoid arthritis, a debilitating chronic systemic autoimmune inflammatory disease [3], orthopaedic conditions, and trauma. It is used to treat painful muscle spasms. It is effective in treating both centrally sourced contractures and reflex types, such as rheumatic and traumatic ones. In addition, THC is used to treat various conditions, such as post-surgical or post-traumatic pain, cervicobrachial neuralgia, acute and chronic torticollis, Parkinson's disease, drug-induced Parkinsonism, and spastic hemiplegia. Sedation was shown to be the primary and most frequently occurring issue with the use of muscle relaxants in normal clinical practice; however, THC was found to not cause sedation in patients receiving continued treatment with it. A common finding in situations of acute lower back pain (LBP) is paravertebral muscular spasms, which can exacerbate the patient's discomfort by setting up a vicious cycle of pain-spasm-pain. THC is frequently used in conjunction with analgesics, NSAIDs, and muscle relaxants as an effective treatment for LBP that occurs acutely. Acute LBP can be effectively treated with THC [4]. It is also used to relieve tooth discomfort [5]. It acts as an anti-inflammatory by suppressing the production of nuclear factor kappa B (NF-κB)-regulated gene products and downregulating them. Terminalia arjuna also shows similar action by inhibiting the cyclooxygenase (COX) enzyme, which hinders the production of prostaglandins [6,7]. Since it works by modulating NF-κB-regulating proteins, it is suggested to be employed as an anti-cancer agent [8].

The European Medical Agency has imposed restrictions on the use of THC, stating that it should not be taken orally for longer than seven days or injected for five days, as a breakdown of the drug in the body may generate the metabolite M2, which can cause aneuploidy. The developing foetus is more severely affected if used during pregnancy. It has also been linked to male infertility [9]. To tackle these shortcomings, formulated eugenol gel can be an alternative, as it provides the required analgesic and anti-inflammatory action and no limitations are put forth for its use [10]. For chronic illnesses, proven herbal remedies are also available that possess the needed anti-inflammatory action, like the bark of *Cinnamomum* *cassia*, whose chemical components prevent the generation of nitric oxide. This is achieved by inhibiting the NF-κB light chain enhancer after it has activated B cells [11].

Although numerous clinical studies eliciting skeletal muscle relaxant activity of THC in low back aches associated with spasms are available [12-17], despite being marketed as a skeletal muscle relaxant for many years, there are few animal studies that demonstrate the skeletal muscle relaxant effect of THC. Thus, it was deemed reasonable to assess the skeletal muscle relaxant activity of THC in an animal model utilizing Wistar rats and a rotarod apparatus.

## Materials and methods

**Figure 1:**
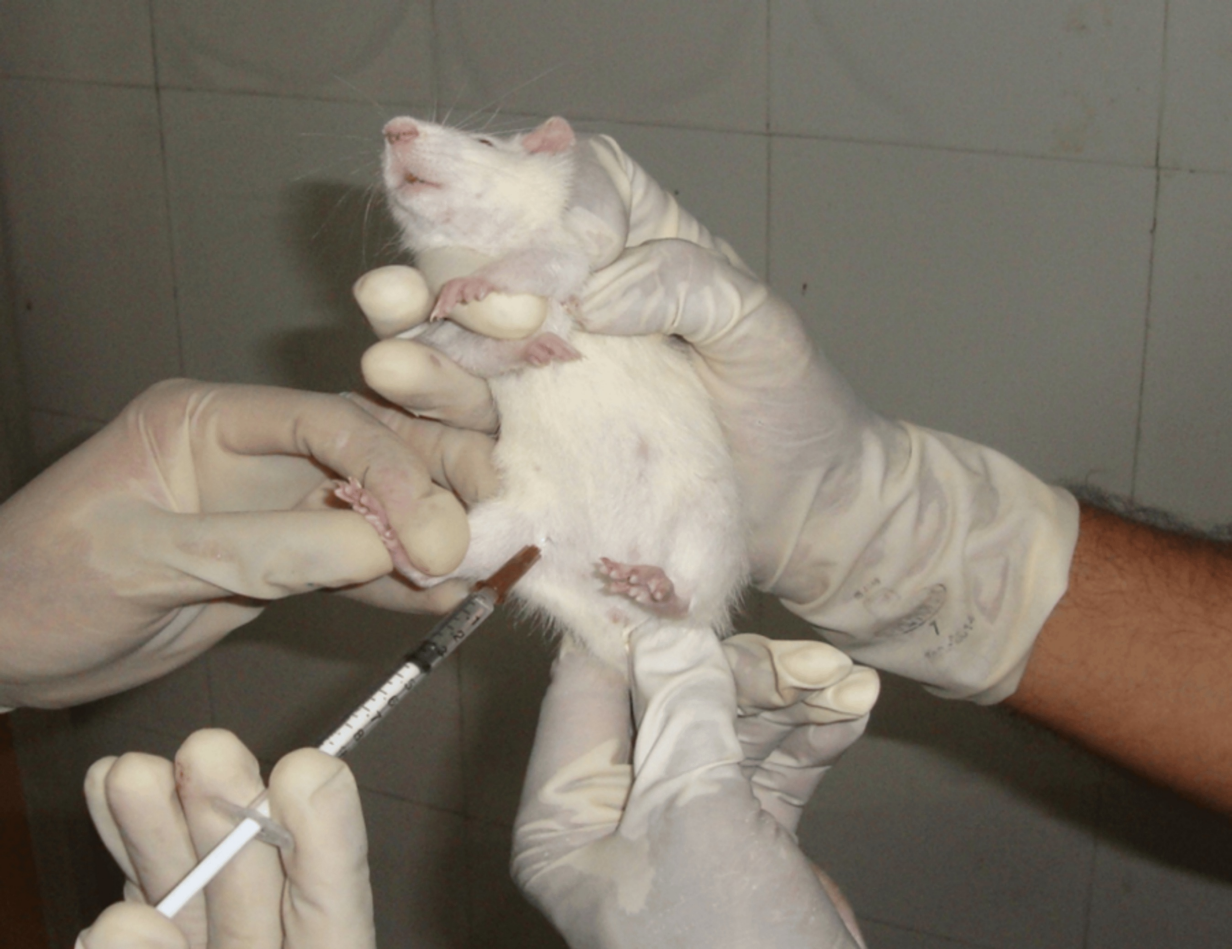
Intraperitoneal injection technique for drug administration

The intraperitoneal (ip) administration technique was used to administer the drugs mentioned above in order to test and compare their skeletal muscle relaxant activity with a standard drug (Figure 1).

**Figure 2:**
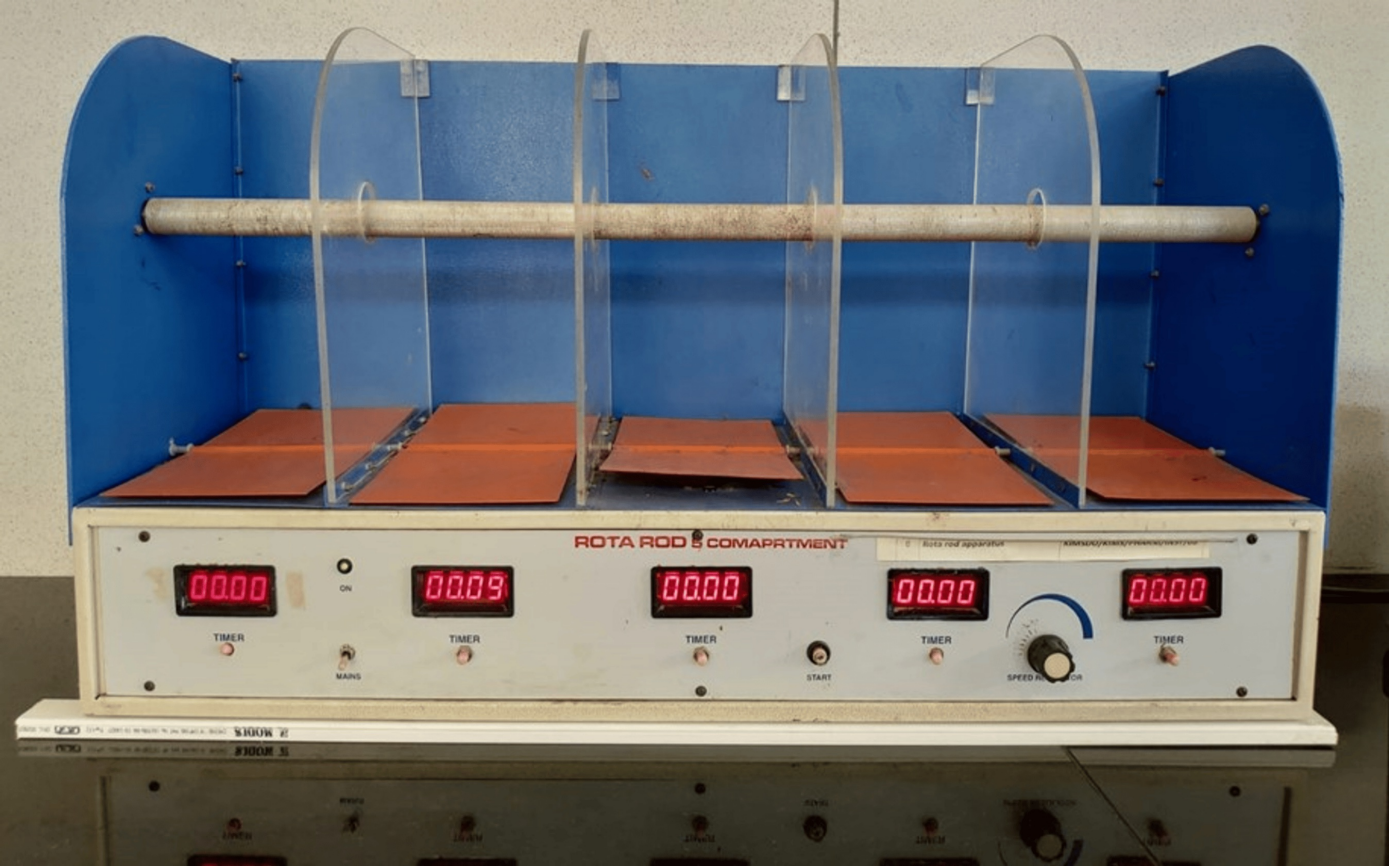
Rotarod apparatus

The rotarod apparatus, used to assess motor coordination of the forelimbs and hindlimbs in rats, was utilized in this study (Figure 2).

Table 1 provides the details on the number of groups, the number of animals per group, the treatment administered to each group, and the abbreviations used for each drug. All drugs were administered ip. A total of 30 Wistar rats were utilized for the study.

**Table 1:** Number of animals and treatment administered mL: millilitre; gm: gram; mg: milligram; kg: kilogram; ip: intraperitoneal; NS: normal saline; THC: thiocolchicoside; DIZ: diazepam

Groups	No. of animals	Treatment	Abbreviation used	Route
I	6	Normal Saline 0.9% 0.1mL/100gm	NS 0.9%	ip
II	6	Thiocolchicoside 2mg/kg	THC 2	ip
III	6	Thiocolchicoside 4mg/kg	THC 4	ip
IV	6	Diazepam 3mg/kg	DIZ 3	ip
V	6	Thiocolchicoside 2mg/kg + Diazepam 3mg/kg	THC 2 + DIZ 3	ip

Experimental animals

Wistar rats, either sex or in good health, weighing 150-180 g, were obtained from the Central Animal House of the Krishna Institute of Medical Sciences. They were housed in suitable conditions with respect to temperature, ventilation, and dietary circumstances. Over the course of the year-long investigation, 30 Wistar rats were used. Rats were acclimated to a 12-hour light-dark cycle over 10 days prior to the experiment day. The animals were brought to the PG research facility of the department and housed there for a minimum of 60 minutes before the experiment to make them adjust to the dim and silent illumination. Every animal was only used once. All observations were taken at 27 and 37 degrees Celsius, respectively, between the hours of 10 am and 5 pm.

Equipments

A 1 mL syringe with a 24 G needle was used for ip drug administration. The rotarod apparatus was used to check the retention time, and a digital weighing machine to measure the weight of the rats according to which dose had to be administered. THC was used as a test drug. It was made available in powdered form. Diazepam (DIZ) was used as a standard drug for comparison with the test drug. It was made available in ampoules. Gloves were used for handling the rats and administering the drugs.

Ethical aspects

The care and usage of laboratory animals were strictly in compliance with the rules established by the Indian National Science Academy (INSA) and the Committee for Control and Supervision of Experiments on Animals (CCSEA). Clearance was given by the Institutional Animal Ethical Committee (IAEC) KIMS, Karad before the experiment commenced (IAEC/KIMS/2021/1). The study was carried out in conformity with the guidelines provided by the Committee for the Purpose of Control and Supervision of Experiments on Animals (CCSEA) at the research lab of the Department of Pharmacology at the Krishna Vishwa Vidyapeeth, Karad.

Chemical drugs and sources

THC was obtained as a pure powder from Indo Phytochem Pvt. Ltd., Kala-Amb, India. The pure powdered form that was used in the study was completely dissolved in water [18], so no solvent was needed. DIZ was purchased from Central Pharmacy, KIMS, Karad. An ampoule of the drug was used in the study. A 0.9% normal saline (NS) solution was utilized as the control in the study.​

Preparation of drugs for injection

THC

An optimal amount of pure powder of a drug and distilled water were used to make a solution for administering the required amount ip. The drug was mixed in water and stirred until it dissolved completely.

DIZ

The required amount of solution was withdrawn from the ampoule and mixed with distilled water to administer the drug ip.

NS

A 0.9% NS was used as a control to compare the skeletal muscle relaxant activity between the drugs. Precautions were taken regarding the volume of injection administered to rats according to the guidelines [19]. Solutions were prepared as per the weight of the rat and the amount of drug to be injected ip.

Experimental model

Rotarod Apparatus

Although the initial rod employed by Dunham and Miya (1957) [20] had a fixed speed, accelerating versions are now available (Jones and Roberts 1968) [21]. The animals were weighed and marked properly. The apparatus has a rubber-coated horizontal metal rod with a diameter of 3 cm that is fastened to the motor. The adjusted speed is two rotations per minute. A 75-cm-long rod is separated into five sections using plastic discs in between them. The height of the rod is kept at 50 cm above the tabletop so that they are unable to escape. The cut-off time is kept at two minutes. At 0 min, 30 min, 60 min, and 120 min, observations were made. Retention time (sec) is recorded [22,23].

Experimental procedure

The inclusion criteria for the study were Wistar rats weighing between 150 and 180 g, regardless of their sex. The exclusion criteria included rats that were pregnant or had recently given birth, rats with a screening time exceeding 120 seconds, and rats exhibiting any enlargements or lesions on their bodies.

Experimental proceedings

The rats were weighed on a digital weighing machine before starting the experiment and put in their respective cages. Cages were labelled as per weight and sex. Precautions were taken that each group remained separately. At 0 min, 30min, 60 min, and 120 min intervals, the respective groups received the drugs ip, as mentioned in Table 1.

Statistical analysis

The data were reported in the format of mean ± standard deviation (SD). The findings of the statistical analysis were obtained using one-way analysis of variance (ANOVA), and the outcomes of the standard drug and control were compared using Dunnett's post-hoc analysis. The asterisk sign indicates statistical significance, with a value of p < 0.05. Version 3.06 of the InStat GraphPad program (GraphPad Software, San Diego, USA) was employed for statistical analysis.

## Results

**Figure 3:**
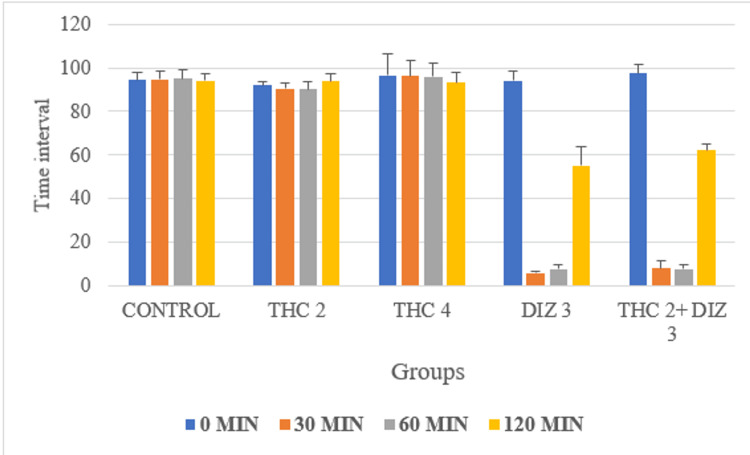
Effects on retention time on rotarod in comparison to control in Wistar rats using THC, DIZ, and combination of THC with DIZ THC: thiocolchicoside; DIZ: diazepam; MIN: minutes

ANOVA showed a statistically significant difference in the retention time over the rotarod in the study groups at all time intervals (THC 2, THC 4, DIZ 3, THC 2 + DIZ 3). As per post hoc analysis by Dunnett's test in comparison with control, a statistically significant difference was seen for DIZ 3 and THC 2 + DIZ 3 at 30 min, 60 min, and 120 min time intervals (p < 0.05). However, there also was a statistically significant difference between THC 2 and THC 4 (p < 0.05) when compared with DIZ (Table 2 and Figure 3).

**Table 2:** Effects of various treatments on retention time on rotarod THC: thiocolchicoside; DIZ: diazepam; min: minutes; sec: seconds; post hoc analysis test: Dunnet's test; p-value: probability value; f-value: ratio of two variances; * statistical significant (p < 0.05)

Time interval (min)	Control (sec) Group I	THC 2 (sec) Group II	THC 4 (sec) Group III	DIZ 3 (sec) Group IV	THC 2+ DIZ3 (sec) Group V	f-value	p-value
0 min	94.6 ± 3.38	92 ± 1.78	96.6 ± 9.81	94.1 ± 4.26	97.5 ± 4.03	0.9715	0.4406
30 min	94.66 ± 3.93	90.5 ± 2.31	96.3 ± 7.44	5.66* ± 0.51	7.83* ± 3.65	739.78	<0.0001
60 min	95 ± 4.05	90.1 ± 3.81	96 ± 6.51	7.66* ± 1.75	7.5* ± 1.97	833.09	<0.0001
120 min	94.16 ± 3.31	93.83 ± 3.37	93.33 ± 4.45	55* ± 8.98	62* ± 2.75	87.19	<0.0001

## Discussion

*Gloriosa Superba L.* seeds are the natural source of THC, a semisynthetic derivative of colchicoside. It is a valuable medicinal plant of industrial importance that is prized for its high colchicine content [24,25]. Currently, discomfort due to low back aches linked to spasms is commonly treated in clinical settings with THC. Its mechanism of action is not clear and is confusing since it has been shown to act on gamma-aminobutyric acid (GABA) type A receptors and glycine receptors, modulate the NF-κB pathway, and COX 2. By acting on muscular contractures and triggering the GABA inhibitory pathways, THC can effectively relax muscles due to its profound and specific sensitivity for type A receptors of GABA [26].

Using a rotarod apparatus as an experimental model, we studied the skeletal muscle relaxant activity of various dosages of THC in the current research. The measure of skeletal muscle relaxant activity was retention time. Every experiment was carried out according to CCSEA requirements. A total of six rats were present in each group. Amongst all the groups, only two treatment groups, DIZ 3 mg/kg, and THC 2 mg/kg + DIZ 3 mg/kg, showed skeletal muscle relaxant activity, and between these two groups, there was no statistically significant difference. This indicates that the addition of THC to DIZ doesn’t potentiate the skeletal muscle relaxant activity of DIZ.

Our results indicated conflicting outcomes in the 2018 study by Sayana et al. on skeletal muscle relaxant activity. They have used nitrazepam (3 mg/kg) and THC (4 mg/kg). It showed THC possesses skeletal muscle relaxant activity in increased doses [27]. In 2001, Tüzün et al. conducted a clinical trial of THC for acute LBP. Their findings contradicted our results. We found that administering THC 4 mg for five days is an effective and safe treatment for patients with acute LBP and muscle spasms [28]. Gervasi et al. conducted a prospective observational study in 2017, which was not consistent with our findings that THC foam helps prevent soreness in the muscles of cyclists [29]. The efficacy and safety of eperisone and THC were compared in patients with acute LBP by Rani et al. who showed better efficacy with eperisone when compared to THC. Their study supported our results [30]. Garg and Yadav conducted a prospective, randomized, open-label trial in 2019 that revealed both tolperisone and THC dramatically decreased discomfort and spasms as well as the distance between the finger and the floor [12].

In 2021, Ketenci et al. conducted a phase IV, multi-centre, randomized, double-blind trial with a parallel group. Their results differ from ours, as they showed that topical THC can be a suitable option for individuals dealing with both muscular spasms and moderate-to-chronic LBP [13]. In 2011, Rao et al. conducted a prospective, randomized, multi-centre, open-label comparative clinical trial, supporting our findings that tolperisone is more effective than THC for individuals experiencing pain-related skeletal muscle spasms [14]. Based on the outcomes of our research, we conclude that THC didn’t show skeletal muscle relaxant activity in Wistar rats in both doses. It has been used and advised for skeletal muscle relaxant activity in humans for many years, especially for myofascial and LBP.

Large-scale clinical studies as well as animal studies pertaining to skeletal muscle relaxant activities using various models of evaluation are needed to prove the usefulness of THC as skeletal muscle relaxant activity. It has to be considered that THC is shown to produce aneuploidy and therefore European Medical Agency has put restrictions on the use of THC clinically. Accordingly, it cannot be used for more than seven days continuously [9]. This study provides scientific proof that THC has no value as a skeletal muscle relaxant in animal studies at the doses used. We have also shown that THC doesn’t potentiate the skeletal muscle relaxant activity of DIZ. Therefore, its use as a skeletal muscle relaxant along with DIZ, baclofen or any other muscle relaxant in a clinical setting might increase the cost of treatment and adverse drug reactions without giving additional muscle relaxant properties because nowadays THC is been administered separately along with DIZ in patients suffering from LBP associated with spasm, thinking that it will potentiate the skeletal muscle relaxant effect of standard drug DIZ.

Limitations

The limitation of our study is the small sample size, as it is an animal-based study. More clinical studies are needed with a large sample size to prove its usefulness and efficacy.

## Conclusions

The current findings of our experimental study led us to the conclusion that THC has not shown skeletal muscle relaxant activity in Wistar rats in both doses of 2 mg/kg body weight and 4 mg/kg body weight. THC doesn’t potentiate the skeletal muscle relaxant activity of DIZ. Considering the adverse effects, restrictions on drug use, and higher cost, studies on the efficacy of the skeletal muscle relaxant on both humans and animals are recommended in order to validate the widespread usage of this drug that is currently being used in clinics.
